# Shaping Policy Change in Population Health: Policy Entrepreneurs, Ideas, and Institutions

**DOI:** 10.15171/ijhpm.2017.143

**Published:** 2018-01-14

**Authors:** Daniel Béland, Tarun R. Katapally

**Affiliations:** ^1^Johnson Shoyama Graduate School of Public Policy, University of Saskatchewan, Saskatoon, SK, Canada.; ^2^Johnson Shoyama Graduate School of Public Policy, University of Regina, Regina, SK, Canada.

**Keywords:** Population Health, Policy Change, Policy Entrepreneurs, Ideas, Institutions, Evidence

## Abstract

Political realities and institutional structures are often ignored when gathering evidence to influence population health policies. If these policies are to be successful, social science literature on policy change should be integrated into the population health approach. In this contribution, drawing on the work of John W. Kingdon and related scholarship, we set out to examine how key components of the policy change literature could contribute towards the effective development of population health policies. Shaping policy change would require a realignment of the existing school of thought, where the contribution of population health seems to end at knowledge translation. Through our critical analysis of selected literature, we extend recommendations to advance a burgeoning discussion in adopting new approaches to successfully implement evidence-informed population health policies.

## Introduction

Population health has evolved as a dominant epidemiological approach that encapsulates principles of both public health and health promotion, and aims to improve the health of the entire population by reducing health inequities between population groups.^1,2^ In essence, the population health approach extends the traditional definition of health (ie, absence of disease) to include one’s capacity to be able to pursue one’s goals, to acquire skills and education, and to grow.^1^ In turn, this approach recognizes that health is influenced by factors beyond healthcare, including political, social, and economic factors and the physical environment,^1,3^ and that the health of populations is influenced by multisectoral policies.

The push for policies that promote health delves into the contentious issue of resource allocation and redistribution across sectors, which would eventually have varied impacts on different populations.^2^ Carrying out population health policies, therefore, requires policy change through critical collaborations across relevant sectors, as well as partnerships between stakeholders in academia, government, health, and healthcare.^5-10^ As Gagnon et al articulate,the implementation of population health policies is further complicated by political realities, institutional structures, and analytical challenges.^11^

Population health advocates need to become more politically astute and pay more attention to political determinants of health.^12^ If population health policies are to be successfully formulated and implemented, we believe that it is imperative to understand how the social science literature on policy change^13^ could be integrated into the population health approach. Here, we set out to examine how key aspects of the policy change literature, especially the work of John W. Kingdon, could contribute towards developing population health policies by facilitating actor mobilization (Policy Entrepreneurs), enhancing flow of policy perspectives (Ideas), and leveraging historically-constructed structures (Institutions) across jurisdictions. Although our article does not review the entire policy change literature, we believe that our focus on policy entrepreneurs, ideas, and institutions offers major insights into how population health scholars and practitioners could better navigate and impact the policy process.

## Policy Entrepreneurs, Ideas, and Institutions

### Policy Entrepreneurs

Policy change is a political reality that is impossible to grasp without paying close attention to human agency^14^ and, more specifically, to the mobilization of specific actors involved in the policy process.^15^ From experts to interest groups, there are many different types of policy actors but, when the time comes to explain policy change, “policy entrepreneur” is a particularly useful analytical category.^16^ Policy entrepreneurs are powerful political and social actors who are in the business of articulating specific problems that move in and out of the policy agenda with new or existing policy solutions. In a specific policy area and at particular points in time, elected officials as well as people associated with political parties, interest groups, international organizations, social movements, and think tanks, can all become policy entrepreneurs as they seek to shape the agenda and promote policy solutions to respond to the perceived economic, social, and environmental problems that influence health of populations.

In fact, according to Kingdon, policy entrepreneurs must take advantage of the short “policy windows” that open in the context of a crisis or a major electoral and political realignment to promote particular solutions to address the policy problems of the day.^17^ Policy entrepreneurs’ “social skills”^18^ are essential to the successful promotion of particular policy solutions within a given “policy window.” Timing is crucial; policy entrepreneurs must be ready to seize the opportunity to promote their preferred policy solution while the “policy window” remains open. For example, when President Obama took office in early 2009, he decided to immediately push for healthcare reform while the Democratic Party controlled both chambers of Congress.^19^

With respect to health policies, although policy entrepreneurs have mobilized to address specific issues (eg, tobacco, homelessness) in multiple countries (eg, Australia, Canada, Sweden),^20-26^ they are still underutilized in population health. The empirical evidence population health research generates can benefit significantly from the incorporation of policy entrepreneurs into population health frameworks/models to increase the probability of evidence-informed policy change.

### Ideas

As Kingdon suggests, policy change is directly impacted by the concrete ideas policy entrepreneurs use to create the political coalitions necessary to bring about change in the context of short “policy windows.”^30^ Policy ideas can take different forms, the most important of which are problem definitions and policy solutions.^31^

First, the ways in which policy problems are defined is crucial for policy change since certain problem definitions are more likely to draw the attention of the public and policy-makers.^32^ More importantly, something must be considered as a collective problem rather than a purely individual matter^32^ for it to make it onto the policy agenda.^33^ For instance, drinking and driving only became perceived as a collective problem worthy of government intervention when policy entrepreneurs mobilized to depict drinking and driving as a social problem rather than a purely individual and moral issue.^35^ This example also illustrates the role of framing in policy change.^36^ This is the case because policy entrepreneurs frame the issues in certain ways to justify public intervention.^37^

Second, policy entrepreneurs can build coalitions around particular policy solutions and pressure government officials to adopt these solutions.^38^ Here, particular ideas such as “solidarity” or “sustainability” can serve as “coalition magnets” that help actors gather around particular policy problems and solutions.^39^ The articulation of these problems and solutions is one of policy entrepreneurs’ major tasks. Policy entrepreneurs must strategically frame issues to bring people together and promote both the adoption and the successful implementation of solutions, which requires bringing state bureaucrats on board by convincing them of the “need to reform.”^40^

### Institutions

While different types of ideas shape policy change, these ideas and the actors who promote them typically exist in a relatively stable institutional environment that creates constraints and opportunities for policy change.^41^ This is why we can supplement Kingdon and his followers’ work on ideas and policy entrepreneurs with a discussion about institutions, which are essential to the comparative analysis of policy change.^42,43^ Two types of institutions are particularly important here: decision-making systems and existing policy legacies.

First, formal political institutions and bureaucratic rules shape political decision-making systems and the role of various policy actors, such as policy entrepreneurs.^43^ Second, existing policy legacies create their own constituencies and vested interests, which tend to support the policy status quo.^44^ At the same time, instead of creating self-reinforcing mechanisms, changing demographic and economic circumstances can generate self-undermining mechanisms that reduce support for them over time as they become increasingly ineffective or expensive.^45^

In federal or decentralized political systems such as the United States, both decision-making systems and existing policy legacies can vary greatly from one state or even one municipality to the next.^46^ This is why the analysis of policy change should take into account how territorially-embedded institutions can shape opportunities for, and obstacles to, policy change. This means that the analysis of policy change at the sub-national level should adopt a comparative lens to grasp key institutional differences among states, regions, or municipalities. Conversely, when moving to the national level, comparisons should be international since comparing the decision-making systems and policy legacies of different countries helps account for why the same policy ideas are successfully adopted and implemented in some countries but not in others.^47^

## Recommendations for Action

### Population Health and Policy Entrepreneurs

At the core of the population health agenda is a philosophy of social justice and equity,^49-51^ which makes population health a politically-charged science where hypotheses are developed based on ideas that lean towards collective good, rather than neo-liberal, free market capitalism.^52^ Although the policy implications of population health are quite clear, population health has largely remained an academic exercise, where frameworks have been developed for examining inequities^53-55^ and complex analyses have focused on generating empirical evidence.^56-58^

Few efforts have been made to leverage this evidence to facilitate policy change, pointing towards an obvious disconnect between generation of empirical evidence and policy formulation and implementation. For example, a significant amount of research has been conducted on the influence of urban planning policy on human health and, although policy initiatives have emerged in response to this research, the rate of translation of evidence into policy does not match the volume of research.^59,60^ The mobilization of policy entrepreneurs over population health issues such as urban planning policy could influence decision makers’ perspective on evidence,^61^ and potentially is a vital missing link in achieving the final goal of effecting policy change based on population health research.

However, we identify three main challenges in developing consensus on advancing the concept of policy entrepreneurs in population health:

1. Broad Scope: Population health encompasses a multitude of disciplines and sectors with research ranging from behavioural sciences to social sciences, and from epidemiology to urban design. This scenario creates the need for both overarching advocacy groups, as well as issue specific (eg, smoking) leaders to act as policy entrepreneurs.
2. Institutional Barriers: Those attempting to create space for new actors will inadvertently face entrenched institutional and systemic barriers. This means that policy entrepreneurs in population health should work with existing networks of researchers, policy-makers, advocates, activists, and non-governmental organizations. Policy entrepreneurs should work closely with existing stakeholders in population health to promote specific policy solutions.
3. Ethical Implications: It is critical to articulate the roles of policy entrepreneurs and population health researchers in effecting policy change to avoid crossing ethical boundaries, especially if the entrepreneurs and researchers are going to benefit from proposed policy changes. For instance, if active researchers work with specific policy entrepreneurs, declaring conflicts of interest would have to be mandatory.

### Shaping Ideas

Population health researchers have a role to play in shaping ideas by utilizing existing tools of knowledge translation^62^ to establish stronger links with policy makers through collaborative scholarship.^63^ Nevertheless, there are limitations to existing knowledge translation approaches in advancing policy change because the theoretical lens in this area focuses exclusively on evidence uptake; rather than view policy change more broadly as a set of ideas driven by competing political forces. As Oliver et al emphasize,^64^ there is a need to generate empirical evidence on the impact of empirical evidence uptake in policy change mechanisms by studying the interaction between population health research and population health policy implementation.

We suggest that the next major effort in moving the population health approach forward would be to develop frameworks for engaging with the politics of policy making. Also, it is time to move beyond knowledge translation to develop ideas that maximize effective use, and minimize misuse, of empirical evidence.^65^ Moreover, if policy entrepreneurs working with researchers are involved as key stakeholders on the path from research to policy change in population health, we think that the probability of shaping ideas based on empirical evidence will increase, as these entrepreneurs can successfully articulate policy problems and provide appropriate policy solutions.

### Understanding Institutions across Jurisdictions

Because both policy change and the mobilization of policy entrepreneurs across jurisdictions are dependent on varying decision-making systems and existing policy legacies,^47^ propagating ideas that could cause policy change should take into account territorially-embedded institutions. Whether it is comprehending institutional differences at the sub-national level (states, regions, and municipalities) or understanding the role of varying decision-making systems in effecting similar policy ideas in different countries,^48^ cross-jurisdictional scans, comparative analyses, and public policy evaluations would be effective tools.^11,66-68^ For example, the imminent legalization of marijuana in Canada across 13 provincial and territorial jurisdictions is bound to raise challenges regarding implementation and regulation.^5,69,70^ This policy change would benefit from cross-jurisdictional scans and comparative analyses that would aid policy entrepreneurs in influencing institutions across jurisdictions.

## Conclusion

Through our critical analysis of relevant literature, especially the work of Kingdon and the concept of policy entrepreneurs, we extend systematic recommendations to advance a burgeoning discussion in adopting new approaches to successfully implement evidence-informed population health policies. Yet the fact that our contribution focuses primarily on Kingdon’s work and the concept of policy entrepreneurs is a clear limitation. More work is needed to engage with, and draw on, other aspects of the rich literature on policy change.

## Acknowledgements

The authors thank Patrik Fafard, Rachel Hatcher, and the anonymous reviewers for their comments and suggestions. Daniel Béland acknowledges support from the Canada Research Chairs Program.

## Ethical issues

Not applicable.

## Competing interests

Authors declare that they have no competing interests.

## Authors’ contributions

The article was co-conceptualized and written by both authors through an iterative process.

## Authors’ affiliations

^1^Johnson Shoyama Graduate School of Public Policy, University of Saskatchewan, Saskatoon, SK, Canada. ^2^Johnson Shoyama Graduate School of Public Policy, University of Regina, Regina, SK, Canada.
